# Irradiated Male Tsetse from a 40-Year-Old Colony Are Still Competitive in a Riparian Forest in Burkina Faso

**DOI:** 10.1371/journal.pone.0037124

**Published:** 2012-05-10

**Authors:** Adama Sow, Issa Sidibé, Zakaria Bengaly, Augustin Z. Bancé, Germain J. Sawadogo, Philippe Solano, Marc J. B. Vreysen, Renaud Lancelot, Jeremy Bouyer

**Affiliations:** 1 Centre International de Recherche-Développement sur l'Elevage en Zone subhumide (CIRDES), Bobo-Dioulasso, Burkina Faso; 2 Pan-African Tsetse and Trypanosomosis Eradication Campaign (PATTEC), Projet de Création de Zones Libérées Durablement de Tsé-tsé et de Trypanosomoses (PCZLD), Bobo-Dioulasso, Burkina Faso; 3 Ecole Inter-Etats des Sciences et Médecine Vétérinaires (EISMV), Dakar-Fann, Sénégal; 4 Institut de Recherche pour le Développement (IRD), UMR 177 IRD-CIRAD INTERTRYP, Montpellier, France; 5 Insect Pest Control Laboratory, Joint FAO/IAEA Programme of Nuclear Techniques in Food and Agriculture, Vienna, Austria; 6 Institut Sénégalais de Recherches Agricoles, Laboratoire National d'Elevage et de Recherches Vétérinaires, Hann, Dakar, Sénégal; 7 UMR Contrôles des Maladies Animales et Emergentes, Centre de Coopération Internationale en Recherche Agronomique pour le Développement (CIRAD), Campus International de Baillarguet, Montpellier, France; National Institute for Communicable Diseases/NHLS, South Africa

## Abstract

**Background:**

Tsetse flies are the cyclical vectors of African trypanosomosis that constitute a major constraint to development in Africa. Their control is an important component of the integrated management of these diseases, and among the techniques available, the sterile insect technique (SIT) is the sole that is efficient at low densities.

The government of Burkina Faso has embarked on a tsetse eradication programme in the framework of the PATTEC, where SIT is an important component. The project plans to use flies from a *Glossina palpalis gambiensis* colony that has been maintained for about 40 years at the Centre International de Recherche-Développement sur l'Elevage en zone Subhumide (CIRDES). It was thus necessary to test the competitiveness of the sterile males originating from this colony.

**Methodology/Principal Findings:**

During the period January–February 2010, 16,000 sterile male *G. p. gambiensis* were released along a tributary of the Mouhoun river. The study revealed that with a mean sterile to wild male ratio of 1.16 (s.d. 0.38), the abortion rate of the wild female flies was significantly higher than before (*p* = 0.026) and after (*p* = 0.019) the release period. The estimated competitiveness of the sterile males (Fried index) was 0.07 (s.d. 0.02), indicating that a sterile to wild male ratio of 14.4 would be necessary to obtain nearly complete induced sterility in the female population. The aggregation patterns of sterile and wild male flies were similar. The survival rate of the released sterile male flies was similar to that observed in 1983–1985 for the same colony.

**Conclusions/Significance:**

We conclude that gamma sterilised male *G. p. gambiensis* derived from the CIRDES colony have a competitiveness that is comparable to their competitiveness obtained 35 years ago and can still be used for an area-wide integrated pest management campaign with a sterile insect component in Burkina Faso.

## Introduction

African animal trypanosomosis (AAT) constitutes a major constraint to livestock production in sub-Saharan Africa. The disease is enzootic in an area covering ca. 10 million km^2^ and threatens nearly 50 million cattle [1]. The disease causes many direct losses due to lower production, mortality and treatment costs, as well as indirect losses such as the opportunity of genetic improvement, and intensification of livestock production [2]. Direct losses and cost of AAT control is estimated to range between USD 600 and 1200 million year^−1^ for sub-Saharan Africa [3].

Tsetse flies are the sole cyclical vectors of trypanosomes, the causative agents of AAT. The maintenance of non-trypanotolerant cattle in tsetse-infested areas is often only feasible through continuous prophylactic and curative treatment with trypanocidal drugs and as a result, more than 35 million doses are being administered annually [4]. Chemoresistance against these drugs is however, becoming more and more widespread [4], [5] making tsetse control the only way to sustainably manage AAT. According to Budd [6], the eradication of trypanosomosis would increase agricultural production in Africa with a value of USD 4.5 billion/year. In addition, tsetse are the vectors of human sleeping sickness, a major neglected disease [7].

Most control tactics against tsetse flies are effective and allow quick reduction of their abundance. The use of insecticide impregnated targets and the application of insecticide pour-ons on cattle reduced tsetse populations in Burkina Faso and in some East African countries drastically [8]–[11] and this was usually followed by a reduction of the AAT incidence [8], [12], [13]. However, these control methods generally do not eradicate the tsetse population because their efficiency is density dependent [14]. Other vector control methods, such as the Sequential Aerosol Technique (SAT), are not tsetse density dependent and can be used to manage tsetse populations in open savannah areas such as *Glossina morsitans centralis* in the Okavango Delta of Botswana [15]. The SAT relies on a high percentage of adult mortality (>99%) during each spraying cycle, which can rarely be attained in the humid or sub-humid areas of West Africa, in view of the dense gallery forests. Finally, the SIT has “negative” density dependent properties, i.e. its efficiency is inversely proportional to the density of the target population because of an increase of the ratio of sterile to wild males at each generation [16]. Therefore, the combination of the SIT (effective at low population densities) with other control techniques that are effective at high population densities is an optimal strategy to achieve eradication of riverine tsetse fly populations in West Africa [17], [18]. However, to warrant a sustainable impact, these tsetse control tactics must be applied area-wide, i.e directed against an entire tsetse population within a delimited area, especially if eradication is the strategy of choice. For example in West-Africa, riverine tsetse species occur in large distribution belts, with established gene flows between the various river basins [19], [20]: their eradication would thus require a sequential approach, including the implementation of barriers between eradication blocks.

Knipling conceived the idea of using sterile insects to manipulate the reproduction rate of a natural insect population in 1937, but it was not until the 1950's that a method was found to sterilize insects and the idea could be given a practical follow-up [21]–[23]. It was for the first time applied in 1954 to eradicate the New World screwworm fly *Cochliomyia hominivorax* (Coquerel) from the Island of Curaçao, Netherlands Antilles [24]. This successful trial was followed by the eradication of the pest from the southern USA, Mexico, Central America (1950–2000) [25], [26], and from Libya in 1990–1991 [27]. Since then, the SIT as part of area-wide integrated pest management (AW-IPM) approaches, has been successfully used to suppress or eradicate several lepidopteran and dipteran pests, including fruit and tsetse flies [16], [28]. It was tested for the first time against *Glossina morsitans morsitans* in Tanzania [29] in the 1970's where sterile males released at a 1.12∶1 ratio managed to maintain the population suppressed for 15 months at a 80–95% reduction level obtained after an initial application of insecticides by air. Thereafter, the release of sterile males was successfully integrated with the deployment of insecticide impregnated targets to eradicate *Glossina palpalis gambiensis* Vanderplank, *Glossina tachinoides* Westwood, and *Glossina morsitans submorsitans* Newstead from an agro pastoral zone of Sidéradougou in Burkina Faso (3,000 km^2^, 1983–1985) and *Glossina palpalis palpalis* Rob. Desv. from a pastoral area in Nigeria (1,500 km^2^, 1982–1985) [18], [30], [31]. Although initially successful, these campaigns in Burkina Faso and Nigeria were not sustainable as the approach was not area-wide (i.e. it did not target the entire tsetse population in a circumscribed geographical area [32]) and the local beneficiary communities and authorities failed to create or maintain adequate buffer areas to prevent re-invasion of the cleared areas [33]. The AW-IPM approach was introduced into the area of tsetse control on the Island of Unguja, Zanzibar, where a population of *Glossina austeni* Newstead was eradicated using the SIT combined with pour-on treatment of cattle and insecticide impregnated targets/screens [34]. The success of this area-wide campaign was a strong argument for the Pan African Tsetse and Trypanosomosis Eradication Campaign (PATTEC), to promote the use of SIT for the eradication of tsetse populations from selected areas in Africa after pre-release reduction of tsetse populations with 90–99% using other effective techniques. In Burkina Faso, more than 20,000 insecticide impregnated targets were deployed along the Mouhoun River and its tributaries to drastically reduce tsetse densities in the PATTEC intervention area. Cypermethrin-based pour-on treatment was applied on cattle in the buffer areas, located at the borders of the study area along 10 km of the Mouhoun River and its tributaries [35]. In addition the eastern branch of the Mouhoun River was treated with the SAT using deltamethrin as active ingredient in collaboration with the PATTEC Ghana office. As a result of this suppression campaign (2009–2010), the populations of *G. p. gambiensis* and *G. tachinoides*, the sole cyclical vectors of trypanosomosis in the area [36], [37], were reduced by 95% in the PATTEC Burkina Faso intervention area [35].

In addition to the mass-rearing of male flies to be sterilized by ionizing irradiation, the SIT can only be successful if these sterile males (i) can locate the wild virgin females and successfully transfer their sterile sperm, and (ii) disperse and aggregate in a similar pattern as their wild counterparts [38]. The Centre International de Recherche-Développement sur l'Elevage en zone Subhumide (CIRDES), Bobo Dioulasso, Burkina Faso has been maintaining a *G. p. gambiensis* colony since 1972, with introduction of wild pupae from time to time. The colony however, has, since the eradication campaign in Sidéradougou in the early 1980's not been used for any operational releases [30]. It is intended to use the sterile flies from this colony for AW-IPM programmes in Burkina Faso and Senegal and it was therefore deemed necessary to re-confirm its field competitiveness [39].

In this study, field releases of sterilized male *G. p. gambiensis* were carried out in riverine gallery forest habitat in Burkina Faso to study their survival, dispersal and aggregation pattern, as well as their mating frequencies with wild female tsetse flies.

## Materials and Methods

### Study area

**Figure 1:**
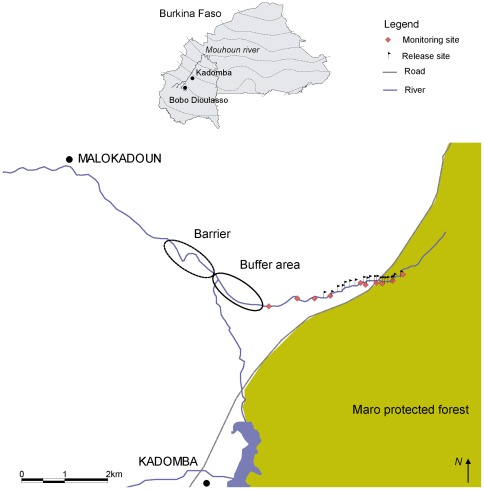
Location of the study area in Burkina Faso. The release and monitoring sites along the Leyessa River are displayed. The 1-km barrier was established with 20 biconical traps impregnated with deltamethrin (see M&M for details).

The study area was situated close to the village of Kadomba (11°53′ North; 3°97′ West), 70 km north of Bobo-Dioulasso (fig. 1) and contained guinean riverine forest [40] along the Leyessa River (a tributary of the Mouhoun River) which has its origin in the protected forest of Maro. Previous entomological surveys showed that almost all caught tsetse were *G. p. gambiensis*, with an average apparent density of 10 flies per trap per day [41]. Laboratory-reared *G. p. gambiensis* were released over 3 km along the river (fig. 1) in geo-referenced release sites. One km upstream of the release area, a 1-km barrier was established with 20 biconical traps impregnated with deltamethrin (800 mg/m^2^) and deployed every ∼50 meters during the whole study. Other studies had revealed that the tsetse population of the study area was genetically differentiated (and thus partially isolated) from that of the Mouhoun River [42].

### Tsetse sterile males

Laboratory-reared tsetse flies used for this study were produced at the CIRDES in Bobo Dioulasso, Burkina Faso. Newly emerged adult male flies were irradiated in a Cs^137^ GAAA ® irradiator with a dose of 110 Gy (dose rate of 4.49 Gy/min), routinely used at CIRDES since the Sideradougou eradication campaign [43]. Irradiated males were marked with acrylic paint on the thorax using different colours to differentiate between series of released insects. Before release, flies were offered twice a blood meal containing isometamidium chloride (Trypamidium® MERIAL SAS, Lyon, France batch nDG/20058) at a concentration of 10 mg/L to prevent the development of trypanosomes in the released flies [44], [45]. Batches of 50 4-day-old male flies were transferred to Roubaud cages (4.5×13×8 cm) that were covered with a net of mesh 1×1 mm. Cages were then put in a humidified container and transported to the release sites.

### Preliminary entomological data collection

Before initiating the field releases, two entomological sampling efforts were carried out during 5 consecutive days, at 10-day intervals. Tsetse flies were sampled with 20 biconical traps [46] deployed at 150 m intervals along the river. All trapping sites were georeferenced. Caught female flies that were still alive were dissected. The same person assessed the percentage of pregnant females and the spermathecal fill during these preliminary sampling periods and during intervention period.

### Release of sterile males

Seven releases were carried out at weekly intervals in January–February 2010. A thousand sterile males were released during each of the first 2 releases, and the number of males released was increased to 2,000 and 4,000 sterile males for the 3 following releases and the last two releases, respectively. A total of 16,000 sterile males were thus released over the 7-week interval, to obtain a ratio of irradiated to wild males upon 1∶1. Releases were made along the river between 4h30 and 6h30 p.m. at equal proportions in 10 different sites interspaced at approximately 300 m. During the releases, dead flies and non-flyers were recorded after opening the Roubaud cages.

### Sterility levels of irradiated male *G. p. gambiensis*

Twenty newly emerged virgin *G. p. gambiensis* females from the CIRDES colony were mated with 10 newly emerged irradiated males and maintained under normal insectary conditions (both males and females were 4 days old). Produced pupae were regularly collected, weighed and stored. Females were dissected after 4 weeks to assess spermathecal fill and their reproductive status. The results were compared to those of a control group of 2000 females the main colony, maintained in the same conditions.

### Dissection of sampled wild female *G. p. gambiensis*

All trapped live female flies were dissected for ovarian ageing to determine the physiological age of the population [47]. Proportions of nulliparous, young (less than 4 ovulations) and old (4 ovulations or more) parous females were determined [48]. Pregnant females were classified as having a larva or a developing egg *in utero*, and non-pregnant females as having an empty uterus. The rate of sterility (natural abortion and induced sterility) was determined taking into consideration the status of the uterus and the follicle next in ovulation sequence: i.e, those females that had recently aborted an egg in embryonic arrest or still had the degenerated egg *in utero*.

The competitiveness of the irradiated males was assessed using the Fried index [49] by comparing the abortion rates obtained during the entomological surveys carried out before, during and after the releases of sterile males. After dissection, spermathecae were placed in a droplet of normal saline solution and were observed under the microscope at 40× magnitude. Spermathecal fill was scored as empty (0) quarter-full (0.25), half-full (0.5), three quarter-full (0.75) and full (1.0) [50], [51].

### Dispersal and population dynamics of the irradiated males

Trapping surveys were implemented weekly after each sterile male release session using 10 biconical traps set along the release area during 2 to 5 days to assess the relative abundance of wild and irradiated males. The colour of the acrylic spot on the thorax indicated the date of release, and thus the age of the trapped flies. The ratio of irradiated to wild male flies in the samples was also calculated.

Recapture of released irradiated males provided an estimate of the number of alive, marked flies for the different series of releases. After the last release, entomological monitoring was continued every week, up to one month after the last release.

### Statistical analyses

**Figure 2:**
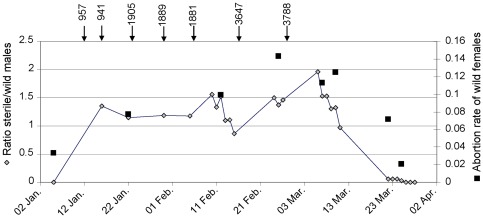
Impact of sterile males releases on wild female abortion rates. Temporal pattern of the ratio of sterile to wild males and the abortion rates of wild females during the study period (Jan.–Apr. 2010) are displayed. The number and dates of sterile flies released are represented by the black arrows at the top.

Mortality rate and survival of the released male flies were estimated using the temporal relative abundance data, assuming a constant daily mortality rate within each released batch. The linear evolution of the captures of the sterile males released on 06/02/10 is presented in fig. 2: this is illustrative of what was observed with other batches. The daily survival rate was thus estimated as the exponential of the slope of the natural logarithm (ln) of total captures for each batch against time.

To assess similarities or differences in the spatial pattern of apparent densities of wild and sterile male flies using trap records, the existence of a spatial trend in log(wild male counts) was tested [38]. This trend was subtracted from log-counts before assessing the independence of trap locations and wild male fly abundance. This was achieved with a Monte Carlo test for marked point processes [52]: the point process being the set of trap locations, and the marks being the wild male fly counts. Secondly, we used a Χ^2^ test to assess the spatial heterogeneity in wild male fly abundance, and correlation tests to assess the independence of wild males and females, and sterile males.

To plot the data, we transformed fly counts into standardized contributions. For each fly category *i* (wild male or female, sterile male) and trap *j* (*j* = 1,… J), each observed trap count *n _i,j_* was divided by the total observed count *N _i_* for this fly category to give the observed relative contribution of each trap *o _i,j_* = *n _i,j_*/*N _i_*. The expected relative contribution of trap *j* under the assumption of homogeneous spatial distribution (*e _i,j_* = 1/*J*) was then subtracted to *o _i,j_* and the result was divided by *e _i,j_*, thus providing the standardized contribution *c _i,j_* = (*o _i,j_*−*e _i,j_*)/*e _i,j_* = *J n _i,j_*/*N _i_*−1.

The proportion of flies having aborted and the spermathecal fill were compared using Χ^2^ tests. A Pearson's correlation test was used to assess the correlation between the ratio of sterile to wild male flies and the abortion rate of wild females, and between the mortality of the sterile males and the number of flies released.

### Ethical statement

All necessary permits were obtained by « Projet de Création de zones libérées durablement de la mouche Tsé-Tsé et de la Trypanosomose » (national project of the Ministry of Animal Resources, N° SAP PZ1-AAO-009), particularly that of the Ministry of Environment of Burkina Faso, which is in charge of the follow up of the environmental impact of this project, for conducting the described field studies as a part of the feasibility study of tsetse eradication in the first block (Mouhoun river loop).

## Results

### Baseline situation

During the two pre-release entomological sampling events, 1950 wild *G. p. gambiensis* were caught giving a relative abundance of 12.2 tsetse flies/trap/day. The sample contained 53.4% female flies of which 166 were dissected. Of these dissected females, 25.9% were nulliparous, 36.1% young and 38% old parous flies. The natural abortion rate was 3.3%, an additional 4.9% of the female flies had an empty uterus (post larviposition) while 39.8% of the female flies contained an egg and 52% a larva.

### Sterile male fly losses during transport

Of the 16,000 irradiated males shipped to the release points, 15,008 (93.8%) were actually released (Table 1), i.e. mortality rate of the male flies at the release sites was 1.9% due to handling, marking, transport or irradiation, and 4.3% were non-flyers and either too weak to take off or had non-functional wings due to acrylic painting.

**Table 1:** Characteristics of the batches of irradiated male *G. p. gambiensis* released in Kadomba.

Date	Colour	Released flies	Flyers (%)	Daily mortality rate (%)	Mean lifespan (days)	Recapture rate (%)
12 Jan	White*	1,000	95.7	10.0	6.60	3.6
16 Jan	Red	1,000	94.1	11.9	5.45	5.6
23 Jan	Yellow	2,000	95.3	16.8	3.76	4.7
30 Jan	Green	2,000	94.4	16.1	3.95	5.0
6 Feb	White*	2,000	94.0	14.7	4.34	15.0
16 Feb	Light red	4,000	91.2	12.5	5.19	4.4
26 Feb	Brillant green	4,000	94.7	22.0	2.79	9.4
Total		16,000	93.8±1.5	14.9±4.0	4.6±1.3	7.2±0.1

* The difference between the released males of these two groups was done by the wing fray interpretation.

### Population dynamics of the irradiated males

**Figure 3:**
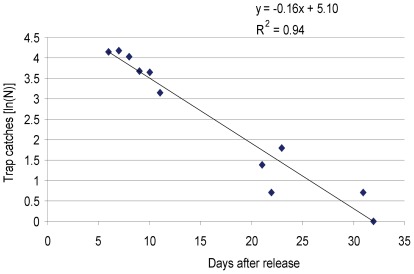
Dynamics of the number of sterile males recaptured. The data correspond to the batch released on 06/02/2010 in Kadomba.

During the monitoring of the dispersal of the irradiated males, 1,068 wild females, 1,048 wild males and 1,142 released males (i.e. 7.6% of the released flies) were trapped. The mean daily mortality rate of the released sterile males was 14.9±4.0%, corresponding to a mean lifespan of 4.6±1.3 days (fig. 3 and table 1). There was no significant correlation between the mortality rate and the number of released flies (p = 0.15). The population of irradiated flies decreased quickly and, one month after the last release no more marked flies were trapped during 3 consecutive days of trap deployment (fig. 2).

### Mating of virgin females with irradiated males

In the laboratory, 48.9% of the colony *G. p. gambiensis* females that had mated with 110 Gy irradiated males had an empty uterus due to abortion, and 6.7% and 44.4% of the female flies had a larva and a degenerating egg *in utero* respectively. The abortion rate was thus higher (p<0.001) than in the control group, where only 1.1%±0.7% of the females aborted. The weight of pupae collected from the different experimental batches was significantly lower (*p*<0.001) than that of pupae produced by the untreated control group i.e. a mean weight of 20.7±2.3 mg vs. 26.8±0.3 mg. Moreover, no adult flies emerged from these pupae. The abortion rate and the spermathecal fill did not vary significantly from one experimental group to another (F = 0.40; df = 6; *p* = 0.75). However, less than one third of females mated with the irradiated males had full spermathecae (31.1%) while 20% had empty spermathecae, which was significantly lower than the mean spermathecal fill of the wild females dissected during the pre-release entomological sampling (χ^2^ = 5.90; *p* = 0.015).

### Competitiveness of irradiated males as compared to wild males

From the second week of sterile male fly releases, the rate of induced sterility increased from 3.3% to 7.7% (s.e. 7.7%) reaching 14% at the end of the releases (fig. 2). It dropped again to 2.1% (s.e. 1.5%) one month after the last release. During the release period, the abortion rate was significantly higher than the natural abortion rate before (n = 338, X2 = 4.932, *p* = 0.026) and after (n = 311, X2 = 5.548, *p* = 0.019) the release period. There was no significant difference between the natural abortion rates recorded before and after the releases (n = 219, X2 = 0.012, *p* = 0.914).

During the entire experiment, the ratio of irradiated to wild males fluctuated between 1 and 2 (fig. 2), and was on average 1.16 (s.d. 0.38). There was a strong positive correlation (*r* = 0.95, *p*<0.001) between the ratio of sterile to wild males and the abortion rate measured during the same week. The competitiveness of the sterile males (Fried index) was 0.07 (s.d. 0.02), corresponding to a sterile to wild male ratio of 14.4 required to obtain 100% induced sterility in female flies.

### Spermathecal fill

During the preliminary fly sampling there was no significant difference between spermathecal fill of the 3 age groups, i.e. teneral/nulliparous, young and old parous wild flies (F = 0.13; df = 2; *p* = 0.88). During the release period, no significant difference was observed between the spermathecal fill of the various age groups (F = 2.05; df = 2; *p* = 0.130). More than 50% of the flies had spermathecae completely filled with sperm and less than 5% had empty spermathecae. Average spermathecal fill of the wild female flies before and after the releases was similar (*p* = 0.149).

### Spatial distribution of the sterile males

Significant spatial trends were observed in the count data: a non-linear (quadratic-like) trend (*p*<10^−4^) was observed for longitude with a maximum close to the eastern region of the study area, and a linear trend (*p*<10^−4^) was observed in latitude, with a maximum in the northern region of the study area.

Similar trends were observed for wild female and sterile male flies, with significant and very similar *p* values. These spatial trends were removed from the data sets for further analyses. The point marked process analysis showed that wild male fly counts were independent from trap locations (Monte Carlo test, *p*>0.05), i.e., no interaction was detected between trap locations and fly counts.

**Figure 4:**
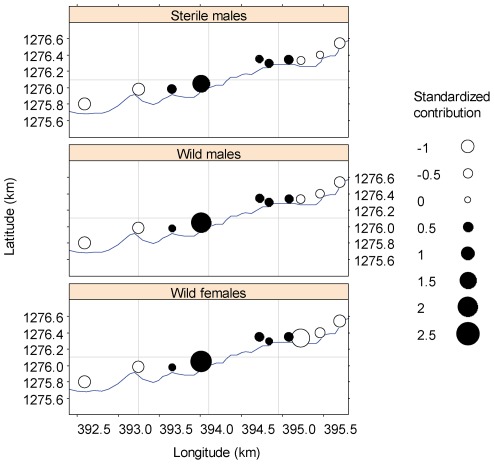
Aggregation patterns of wild and sterile *Glossina palpalis gambiensis*. The figure displays the spatial distribution (standardized abundance) of wild and sterile tsetse sampled with 10 biconical traps along the Leyessa River. See text for explanations on standardized abundance. Longitude and latitude are expressed according to the coordinate reference system UTM 30N, Clarke 1880 ellipsoid.

Although sterile male flies were released rather homogeneously along the river (fig. 1, mean distance between release sites = 102 m, s.d. = 49 m) their spatial distribution of recapture was highly heterogeneous, as evidenced by trapping counts with spatial trend removed (Χ^2^ = 34, df = 9, *p* = 10^−4^). The distributions of male and female wild fly catches were also heterogeneous (Χ^2^ = 133, df = 9, p<10^−4^; Χ^2^ = 25, df = 9, *p* = 0.003). The joint distribution of these de-trended counts is shown in Figure 4. The spatial distribution of sterile and wild male fly catches was similar (*p* = 0.94).

## Discussion

The use of sterile insects as part of AW-IPM can only be successful if the male flies released are of the highest biological quality. They should have adequate survival, intermingle with the wild insect population and be capable of transferring their sterile sperm to virgin females preferably in the same frequency as their natural male counterparts. The competitiveness of sterile insects becomes the more questionable when they have been colonised for multiple generations, as is the case with the *G. p. gambiensis* colony maintained at the CIRDES in Burkina Faso. Recently, it was demonstrated that this strain was still competitive with two field strains originating from Mali and Senegal in experimental conditions [53]. The results of these experimental releases clearly indicate that the competitiveness and behaviour of irradiated male *G. p. gambiensis* derived from the CIRDES colony under field conditions was comparable with data obtained 30 years ago.

The percentage of flies actually released (94%) as a proportion of total flies transported, was satisfactory and similar to that obtained by Clair *et al.* (1976) in Burkina Faso [54]. Mortality rates before release and the proportion of non-flying alive flies in our experiment were very close to their observations, indicating adequate handling, irradiation, transport, and release procedures. Although the average daily mortality rate after release can be considered as high i.e. 14.9% and corresponding to a mean lifespan of 4.6 days, these data are in line with results obtained from January to March 1984 during the Sidéradougou eradication campaign using flies derived from the same colony [30], [31]. A re-analysis of the raw data available during this period (Bouyer J., unpublished) revealed a daily mortality of 13.6%, s.d. 6%. However, during the rainy season of the same year (August–October 1984) a much lower daily mortality rate of 9% (s.d. 3%) was observed. The high mortality rates in our experiment could therefore be attributed to the hot dry season in the area when temperatures were high and the stress on the sterile flies considerable. It would be useful to expand this study and assess performance of the sterile males during the rainy season. The mean lifespan obtained during our study is also close to the one obtained with irradiated males of *G. tachinoides* released around N'Djamena (Chad) in 1973 (4.8 days) [55]. Generally, the longevity of irradiated male tsetse flies is reduced as compared with wild flies due to (i) successive anaesthesia using cold temperatures, (ii) handling in the insectary for sorting and marking, and (iii) irradiation using ionizing radiation that inflicts somatic cell damage [56]. Former studies in the laboratory showed that mean longevity was considerably reduced in irradiated *G. tachinoides*, *G. fuscipes fuscipes* Newstead, and *G. brevipalpis* [57]. These shorter lifespans must be compensated for by regular (twice weekly) releases during an eradication campaign to maintain critical sterile to wild male overflooding ratios [58]. In the case of tsetse, the shorter lifespan presents however an advantage as it reduces the risk of sterile males transmitting the trypanosomoses [44], [45].

The spatial analysis showed that the observed heterogeneous fly distribution among traps was not related to differences in trap efficiency, but to a fly aggregation in preferred sites (and fly emigration from other sites) as was observed on Unguja Island (Zanzibar) during the eradication campaign of *G. austeni* [38]. Barclay [59] has shown the importance of insect aggregation in pest control, especially when using the SIT or any other genetic control method. The weaker correlation between wild males and females, and absence of correlation between sterile males and wild females might be related to different, sex-specific preferences in fly habitat, as observed on Unguja Island [38]. In addition, the present data confirm that tsetse fly dispersal cannot be solely considered as a homogeneous diffusion process, as often assumed [60], [61]. It also confirms that mass-reared and gamma-sterilized male tsetse flies were able to respond to environmental cues and to aggregate in the preferred sites of the wild males, even after being colonised for about 40 years. Their aggregation behaviour was therefore similar to that of wild males, confirming that sterile male flies derived from the CIRDES colony are still very good candidates for genetic control.

The competitiveness of irradiated males was assessed through the comparison of the abortion rate and spermathecal fill before, during and after the releases of irradiated males, and the analysis of their spatial distribution, as compared with their wild counterparts. During the collection of initial baseline data, the observed natural abortion rate of 3.3% was close to the rate of natural abortion noted in *G. austeni* [34]. Under adverse climatic conditions, the abortion rate in female tsetse can reach 9%, as observed in *G. m. morsitans* in Zambia [62]. However, there was no climatic accident during the study, but instead a progressive increase of the mean temperature from 27.6°c to 32.6°c and of the mean relative hygrometry from 16.6% to 35.8% (data not shown), whereas the abortion rate dropped again after the release period. The low spermathecal fill observed in laboratory cage conditions in comparison to the wild females is probably related to the use of 4 day-old males, which are not as competitive as older males. During 11 weeks of monitoring, the ratio of irradiated/wild males was on average 1.16 (s.d. 0.38). These results confirmed that 110 Gy-irradiated, previously fed male *G. p. gambiensis* disperse well and have a significant impact on the reproduction of wild females. Obviously, such a low release rate would neither allow a significant induction of sterility in the wild female population or/and a significant reduction of the wild tsetse population in the release area. In our experiment, the wild *G. p. gambiensis* population was deliberately not suppressed before release of sterile male flies because we needed an adequate number of wild female flies for dissection to assess the impact of sterile males on their sterility. The data of the experiment clearly indicated that irradiated male *G. p. gambiensis* reared at CIRDES are still compatible with the wild females and competitive with the wild males in the Mouhoun River basin. This is probably due to the slow reproductive cycle of tsetse (one offspring every 10 days resulting in a mean number of only 4.7 pupae by female in the insectarium of CIRDES), which imposes relatively low selection pressures on flies maintained in a colony. This makes it necessary to keep a large colony to produce the sterile males, as it was done in CIRDES (between 50 000 and 100 000 females during the last 20 years), thus allowing to maintain the genetic diversity.

Within the framework of the eradication project in Burkina Faso, it will however be necessary to overflood the wild male population with a higher ratio (>14.4). That is why pre-release suppression by other effective techniques (target, pour on treatment of cattle, SAT and ground fogging) will be implemented in the Mouhoun River basin before using the SIT as was done in the 1980's in the Sidéradougou area [30], [31].

## References

[1] Kristjanson PM, Swallow BM, Rawlands GJ, Kruska RL, Leeuw PN (1999). Measuring the cost of African Animal Trypanosomiasis, the potential benefits of control and returns to research.. Agr syst.

[2] Shaw APM, Maudlin I, Holmes PH, Miles MA (2004). Economics of African trypanosomosis.. The trypanosomosis.

[3] Swallow BM (1999). Impacts of Trypanosomiasis on African Agriculture..

[4] Geerts S, Holmes PH, Diall O, Eisler MC (2001). African bovine trypanosomiasis: the problem of drug resistance.. Trends Parasitol.

[5] Delespaux V, Geysen D, Van Den Bossche P, Geerts S (2008). Molecular tools for the rapid detection of drug resistance in animal trypanosomes.. Trends Parasitol.

[6] Budd L (1999). DFID-funded tsetse and trypanosome research and development since 1980..

[7] Simarro PP, Cecchi G, Paone M, Franco JR, Diarra A (2010). The Atlas of human African trypanosomiasis: a contribution to global mapping of neglected tropical diseases.. Int J Health Geogr.

[8] Bauer B, Amsler-Delafosse S, Kaboré I, Kamuanga M (1999). Improvement of cattle productivity through rapid alleviation of African Trypanosomosis by integrated disease management practices in the Agropastoral zone of Yalé, Burkina Faso.. Trop Anim Health Prod.

[9] Bauer B, Amsler-Delafosse S, Clausen P, Kabore I, Petrich-Bauer J (1995). Successful application of deltamethrin pour on to cattle in a campaign against tsetse flies (*Glossina* spp.) in the pastoral zone of Samorogouan, Burkina Faso.. Trop Med Parasitol.

[10] Hargrove JW, Torr SJ, Kindness HM (2003). Insecticide-treated cattle against tsetse (Diptera: Glossinidae): what governs success?. Bull Entomol Res.

[11] Kagbadouno MS, Camara M, Bouyer J, Courtin F, Morifaso O (2011). Tsetse control in Loos islands, Guinea.. Parasites & Vectors.

[12] Rowlands GJ, Leak SGA, Mulatu W, Nagda SM, Wilson A (2000). Use of deltamethrin ‘pour-on’ insecticide for the control of cattle trypanosomosis in the presence of high tsetse invasion.. Med Vet Entomol.

[13] Bouyer J, Stachurski F, Gouro A, Lancelot R (2009). Control of bovine trypanosomosis by restricted application of insecticides to cattle using footbaths.. Vet Parasitol.

[14] Bouyer J, Solano P, Cuisance D, Itard J, Frézil J-L, Lefèvre P-C, Blancou J, Chermette R, Uilenberg G (2010). Trypanosomosis: Control methods.. Infectious and parasitic diseases of livestock.

[15] Kgori PM, Modo S, Torr SJ (2006). The use of aerial spraying to eliminate tsetse from the Okavango Delta of Botswana.. Acta Trop.

[16] Dyck VA, Hendrichs J, Robinson AS (2005). Sterile insect technique..

[17] Vloedt van der AMV, Baldry DAT, Politzar H, Kulzer H, Cuisance D (1980). Experimental helicopter applications of decamethrin followed by release of sterile males for the control of riverine vectors of trypanosomiasis in Upper Volta.. Insect Sci Applic.

[18] Takken V, Oladunmade MA, Dengwat L, Feldmann HU, Onah JA (1986). The eradication of Glossina palpalis palpalis (Robineau-Desvoidy) (Diptera: Glossinidae) using traps, insecticide-impregnated targets and the sterile insect technique in central Nigeria.. Bull Entomol Res.

[19] Bouyer J, Ravel S, Guerrini L, Dujardin JP, Sidibé I (2010). Population structure of *Glossina palpalis gambiensis* (Diptera: Glossinidae) between river basins in Burkina-Faso: consequences for area-wide integrated pest management.. Inf Gen Evol.

[20] Koné N, Bouyer J, Ravel S, Vreysen MJB, Domagni KT (2011). Contrasting Population Structures of Two Vectors of African Trypanosomoses in Burkina Faso: Consequences for Control.. PLoS Negl Trop Dis.

[21] Knipling EF (1955). Possibilities of insect population control through the use of sexually sterile males.. J Econ Entomol.

[22] Klassen W (2003). Edward F. Knipling: Titan and driving force in ecologically selective area-wide pest management.. J Am Mosq Control Assoc.

[23] Knipling EF (1959). Sterile-Male Method of Population Control: Successful with some insects, the method may also be effective when applied to other noxious animals.. Science.

[24] Baumhover AH, Graham AJ, Bitter BA, Hopkins DE, New WD (1955). Screwworm control through release of sterile flies.. J Econ Entomol.

[25] Vargas-Terán M (1991). The New World Screwworm in Mexico and Central America..

[26] Novy JE (1991). Screwworm control and eradication in the southern United States of America..

[27] Vargas-Terán M, Hursey BS, Cunningham EP (1994). Eradication of the screwworm from Libya using the sterile insect technique.. Parasitol Today.

[28] Dagnachew S, Sangwan AK, Abebe G (2005). Epidemiology of Bovine Trypanosomosis in the Abay (Blue Nile) Basin Areas of Northwest Ethiopia.. Rev Elev Méd vét Pays trop.

[29] Williamson DL, Baumgartner HM, Mtuya AG, Warner PV, Tarimo SA (1983). Integration of insect sterility and insecticides for control of Glossina morsitans morsitans (Diptera: Glossinidae) in Tanzania: I. Production of tsetse flies for release.. Bull Entomol Res.

[30] Cuisance D, Politzar H, Merot P, Tamboura I (1984). Les lâchers de mâles irradiés dans la campagne de lutte intégrée contre les glossines dans la zone pastorale de Sidéradougou, Burkina Faso.. Rev Elev Méd vét Pays trop.

[31] Politzar H, Cuisance D (1984). An integrated campaign against riverine tsetse flies *Glossina palpalis gambiensis* and *Glossina tachinoides* by trapping and the release of sterile males.. Insect Sci Applic.

[32] Vreysen M, Robinson AS, Hendrichs J (2007). Area-Wide Control of Insect Pests, From research to field implementation..

[33] Sow A, Sidibé I, Bengaly Z, Bouyer J, Bauer B (2010). Fifty years of research and fight against tsetse flies and animal trypanosomosis in Burkina Faso. An overview.. Bull Anim Hlth Prod.

[34] Vreysen MJB, Saleh KM, Ali MY, Abdulla AM, Zhu Z-R (2000). *Glossina austeni* (Diptera: Glossinidae) Eradicated on the Island of Unguja, Zanzibar, Using the Sterile Insect Technique.. J Econ Entomol.

[35] Pan-African Tsetse, Trypanosomosis Eradication Campaign (PATTEC)/Projet de Création de Zones Libérées Durablement de Tsé-tsé et de Trypanosomoses (PCZLD) (2010). Bobo-Dioulasso, Burkina Faso.

[36] Bouyer J, Guerrini L, Desquesnes M, de la Rocque S, Cuisance D (2006). Mapping African Animal Trypanosomosis risk from the sky.. Vet Res.

[37] Van den Bossche P, de La Rocque S, Hendrickx G, Bouyer J (2010). A changing environment and the epidemiology of tsetse-transmitted livestock trypanosomiasis.. Trends Parasitol.

[38] Vreysen MJB, Saleh KM, Lancelot R, Bouyer J (2011). Factory tsetse flies must behave like wild flies: a prerequisite for the sterile insect technique.. PLoS Negl Trop Dis.

[39] Bouyer J, Seck MT, Sall B, Guerrini L, Vreysen MJB (2010). Stratified entomological sampling in preparation of an area-wide integrated pest management programme: the example of *Glossina palpalis gambiensis* in the Niayes of Senegal.. J Med Entomol.

[40] Bouyer J, Guerrini L, César J, de la Rocque S, Cuisance D (2005). A phyto-sociological analysis of the distribution of riverine tsetse flies in Burkina Faso.. Med Vet Entomol.

[41] Koné N, N'Goran EK, Sidibé I, Kombassere AW, Bouyer J (2011). Spatio-temporal distribution of tsetse (Diptera: Glossinidae) and other biting flies (Diptera: Tabanidae and Stomoxinae) in the Mouhoun River Basin, Burkina Faso.. Med Vet Entomol.

[42] Esnault O (2007). Etude de la structuration de deux sous-populations de Glossina palpalis gambiensis Vanderplank sur la Leyessa, un affluent du Mouhoun au Burkina Faso..

[43] Taze Y, Cuisance D, Politzar H, Clair M, Sellin E (1977). Essais de détermination de la dose optimale d'irradiation des mâles de *Glossina palpalis gambiensis* (Vanderplank, 1949) en vue de la lutte biologique par lâchers de mâles stériles dans la région de Bobo-Dioulasso (Haute Volta).. Rev Elev Méd vét Pays trop.

[44] Van den Bossche P, Akoda K, Djagmah B, Marcotty T, De Deken R (2006). Effect of Isometamidium Chloride Treatment on Susceptibility of Tsetse Flies (Diptera: Glossinidae) to Trypanosome Infections.. J Med Entomol.

[45] Bouyer J (2008). Does isomethamidium chloride treatment protect tsetse flies from trypanosome infections during SIT campaigns?. Med Vet Entomol.

[46] Challier A, Laveissière C (1973). Un nouveau piège pour la capture des glossines (*Glossina:* Diptera, Muscidae): description et essais sur le terrain.. Cah ORSTOM, sér Ent Méd et Parasitol.

[47] Challier A (1965). Amélioration de la méthode de détermination de l'âge physiologique des glossines.. Bull Soc Path Ex.

[48] Laveissière C, Grébaut P, Herder S, Penchenier L (2000). Les glossines vectrices de la Trypanosomiase humaine africaine; IRD, editor..

[49] Fried M (1971). Determination of Sterile-Insect Competitiveness.. J Econ Entomol.

[50] Pollock JN (1982). Training Manual for Tsetse Control Personnel..

[51] Abila PP, Kiendrebeogo M, Mutika GN, Parker AG, Robinson AS (2003). The effect of age on the mating competitiveness of male *Glossina fuscipes fuscipes* and *G. palpalis palpalis*.. J Insect Sci:.

[52] Schlather M, Ribeiro PJ, Diggle PJ (2004). Detecting dependence between marks and locations of marked point processes.. J Roy Stat Soc Ser B (Stat Method).

[53] Mutika GN, Kabore I, Seck MT, Sall B, Bouyer J (2012). Mating performance of *Glossina palpalis gambiensis* strains from Burkina Faso, Mali and Senegal..

[54] Clair M, Politzar H, Cuisance D, Lafaye A (1976). Observations sur un essai préliminaire de lâchers de mâles stériles de *Glossina palpalis gambiensis* (Haute-Volta).. Rev Elev Méd vét Pays trop.

[55] Cuisance D, Itard J (1973). Comportement de mâles stériles de Glossina tachinoides West. lâchés dans les conditions naturelles – environs de Fort-Lamy (Tchad). II. Longévité et dispersion.. Rev Elev Méd vét Pays trop.

[56] Vreysen MJB (2001). Principles of area-wide integrated tsetse fly control using the Sterile Insect Technique.. Med Trop.

[57] Vreysen MJB, Van der Vloedt AMV, Barnor H (1996). Comparative gamma radiation sensitivity of *G. tachinoides* Westw., *G. f. fuscipes* Newst., and *G. brevipalpis* Newst.. Int J Radiat Biol.

[58] Hendrichs J, Vreysen MJB, Enkerlin WR, Cayol JP, Dyck VA, Hendrichs J, Robinson AS (2005). Strategic Options in Using Sterile Insects for Area-Wide Integrated Pest Management.. Sterile insect technique.

[59] Barclay HJ (1992). Modelling the effects of population aggregation on the efficiency of insect pest control.. Res Pop Ecol.

[60] Bouyer J, Balenghien T, Ravel S, Vial L, Sidibé I (2009). Population sizes and dispersal pattern of tsetse flies: rolling on the river?. Mol Ecol.

[61] Hargrove JW (2000). A theoretical study of the invasion of cleared areas by tsetse flies (Diptera: Glossinidae).. Bull Entomol Res.

[62] Challier A (1982). The ecology of tsetse (*Glossina* spp.)(Diptera, Glossinidae): a review.. Insect Sci Applic.

